# Activation of astrocyte Gq pathway in hippocampal CA1 region attenuates anesthesia/surgery induced cognitive dysfunction in aged mice

**DOI:** 10.3389/fnagi.2022.1040569

**Published:** 2022-11-11

**Authors:** Xupeng Wang, Yanan Li, Juan Zhao, Jiaxu Yu, Qi Zhang, Fang Xu, Yahui Zhang, Qi Zhou, Chunping Yin, Zhiyong Hou, Qiujun Wang

**Affiliations:** ^1^Department of Anesthesiology, Third Hospital of Hebei Medical University, Shijiazhuang, Hebei, China; ^2^Teaching Experiment Center, Hebei Medical University, Shijiazhuang, Hebei, China; ^3^Department of Anesthesiology, Hebei Children’s Hospital Affiliated to Hebei Medical University, Shijiazhuang, Hebei, China; ^4^Department of Orthopedics, The Third Hospital of Hebei Medical University, Shijiazhuang, Hebei, China; ^5^NHC Key Laboratory of Intelligent Orthopaedic Equipment, The Third Hospital of Hebei Medical University, Shijiazhuang, Hebei, China

**Keywords:** POCD, astrocyte, synaptic plasticity, chemical genetics, aged mice

## Abstract

The elderly are particularly vulnerable to brain dysfunction after fracture surgery, but the mechanism underlying the cognitive decline due to anesthesia/surgery is not well understood. In this study, we observed hippocampus-dependent cognitive impairment in aged mice undergoing anesthesia and tibial fracture surgery, a common model of postoperative cognitive dysfunction in aged mice. We used Golgi staining and neuroelectrophysiological techniques to detect structurally and functionally impaired synaptic plasticity in hippocampal CA1 region of Postoperative cognitive dysfunction aged mice, respectively. Based on the ‘third party synapse’ hypothesis of astrocytes, we used glial fibrillary acidic protein to label astrocytes and found an increase in abnormal activation of astrocytes in the CA1 region of hippocampus. We hypothesize that abnormal astrocyte function is the driving force for impaired synaptic plasticity. So we used chemogenetic methods to intervene astrocytes. Injection of adeno-associated virus into the CA1 region of the hippocampus bilateral to aged mice resulted in the specific expression of the Gq receptor, a receptor specially designed to be activated only by certain drugs, within astrocytes. The results of novel object recognition and conditioned fear experiments showed that CNO activation of astrocyte Gq pathway could improve the learning and memory ability and the synaptic plasticity of Postoperative cognitive dysfunction aged mice was also improved. The results of this study suggest that activation of the Gq pathway in astrocytes alleviates Postoperative cognitive dysfunction induced by anesthesia and surgery in aged mice.

## Introduction

With the continuous improvement and development of the economy and medical system, the quality of life has been improved and the life expectancy has been extended (Kontis et al., 2017). Aging is a common phenomenon in our society, and the number of fractures in this population is increasing every year due to the deterioration of physical abilities, weakness and osteoporosis (Gallagher, 2004; Frisoli et al., 2021). The incidence of postoperative cognitive dysfunction (POCD) is very high in elderly patients after fracture, surgery and anesthesia (Papadopoulos et al., 2014; Beishuizen et al., 2017; Kristek et al., 2019). POCD is one of the most common postoperative complications in elderly patients, manifested by cognitive decline, anxiety, reduced attention, impaired memory, impaired language comprehension and social integration. A growing body of evidence from clinical studies suggests that POCD may lead to longer hospital stays, reduced quality of life, and increased mortality (Holmgaard et al., 2019; Lin et al., 2020). However, the mechanism of POCD in elderly patients remains unclear. How to provide a stable perioperative period for elderly patients and protect their cognitive function from being impaired has become an urgent clinical problem. Numerous studies have shown that the hippocampal CA1 region is critical for recent memory and that the occurrence of POCD is associated with altered synaptic plasticity of neurons in the hippocampal CA1 region (Tian et al., 2015; Xiao et al., 2018; Liu et al., 2021). The exact mechanism is subject to different opinions. Astrocytes are the most numerous non-neural cells in the brain and are known to provide structural and metabolic support for neuronal networks, but there is growing evidence that they also play an active role in regulating neuronal activity (Suzuki et al., 2011; Zhou et al., 2021). Recent studies have shown that astrocytes are required for synaptic plasticity and normal memory capacity, and have even proposed the hypothesis of “tripartite synapses”-that astrocytes not only wrap, support, and insulate synapses, but also sense and actively modify synaptic structures (Durkee and Araque, 2019). Chemical genetic techniques based on engineered proteins are a powerful approach featuring chemical genetic techniques for designing receptors exclusively activated by designer drugs (DEARD; Gomez et al., 2017). Many recent studies have used astrocyte-specific chemogenetic techniques to confirm the importance of activation of astrocytes in memory formation. For example, Adamsky et al. (2018) demonstrated that activation of astrocytes led to increased spontaneous vesicle release and NMDA-mediated *de novo* synaptic potentiation. Suzuki et al. (2011) reported a key role of astrocytes in the transition from short-term to long-term memory. However, most studies on astrocytes have focused on their positive role in normal memory, and relatively few studies have been conducted on the role of astrocytes in POCD. In the present study, we found abnormal activation of astrocytes in the hippocampal CA1 region of POCD-aged mice. By injecting adeno-associated virus expressing specially designed receptor hM3D under the control of glial fibrillary acidic protein promoter in the hippocampal CA1 region, the cognitive decline in aged mice could be improved, revealing that abnormal astrocyte function may be one of the causes of POCD in aged mice, and activation of astrocyte Gq-GPCR signaling in the hippocampal CA1 region could improve postoperative cognitive dysfunction.

## Materials and methods

### Animals

A total of 55 18-month-old, weighing 25–32 g, specific pathogen-free (SPF)-grade male C57BL/6 J mice purchased from Hebei Ex&InVivo Biotechnology Co., Ltd. [License No.: SCXK(J)2020–002] were used in this experiment. Two mice were excluded because anatomical repositioning was not achieved, and another three were excluded because the cerebral cortex was damaged during cranial drilling. All excluded mice were replenished afterwards, resulting in 10 mice per group (n = 10). All mice were housed in an animal room with temperature maintained at (25 ± 1) °C, humidity at (55 ± 5) %, and a light/dark cycle of 12 h/12 h. The Ethics Committee of the Third Hospital of Hebei Medical University has approved the experimental design and protocol [GA2017-026-1]. All mice were habituated in separate cages for 1 week prior to surgery, and all mice received human care during the experiments with reference to the recommendations of the Guide for the Care and Use of Laboratory Animals published by the National Institutes of Health.

### Anesthesia/surgical model

**Figure 1:**
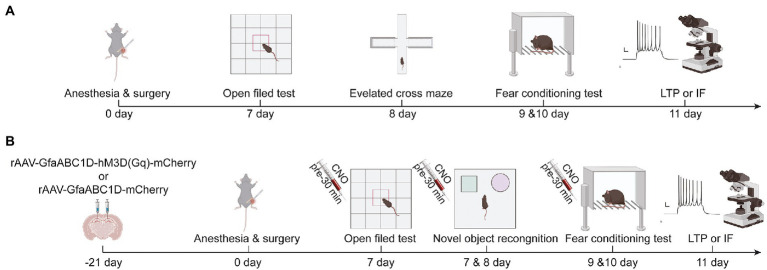
**(A)** Schematic diagram of experimental procedure of aging mice in group C and TF, **(B)** Schematic diagram of experimental procedure of aging mice in AAV mCherry/CNO group, AAV hM3D/Saline group and AAV hM3D/CNO group. Images were created on the BioRender web platform.

Anesthesia/surgery model was established by performing right tibial fracture surgery on mice under sevoflurane anesthesia as described in other studies (Liu et al., 2021). In the first experiment, mice were randomly divided into two groups (*n* = 10): the control group (C) and the tibial fracture surgery group (TF). Mice in group C did not receive anesthesia or any surgical stimulation. Older mice in the TF group were placed in an anesthesia-inducing chamber pre-filled with 5% sevoflurane (21,070,531, Shanghai Hengrui Pharmaceutical Co., Ltd., China, Shanghai) and removed after the righting reflex disappeared. They were then fixed in the left lateral position on a heated blanket-lined operating table and maintained under anesthesia with 3% sevoflurane. To provide good intra-and postoperative analgesia, the incision site was infiltrated anesthesia with 0.5% lidocaine after disinfecting and removing the hair of the surgical site. A longitudinal incision of approximately 1 cm was subsequently made along the medial surface of the tibia to fully expose the tibial plateau. An 8-mm long needle with an internal diameter of 0.3 mm was inserted vertically into the tibial plateau and along the longitudinal axis of the tibial marrow cavity, and then the upper middle part of the tibia was cut with a scalpel. Finally, the incision was sutured, and the mice were kept warm and allowed to awaken naturally. The total time was about 20 min (Figure 1A).

### Stereotactic injection of virus

In the interventional study, mice were randomly divided into 3 groups (n = 10): the TF + rAAV5-GfaABC1D-mCherry + CNO group (AAV-mCherry/CNO), the TF + rAAV5-GfaABC1D-hM3D(Gq)-mCherry + saline group (AAV-hM3D/Saline), and the TF + rAAV5-GfaABC1D-hM3D(Gq)-mCherry + CNO group (AAV-hM3D/CNO). The experimental procedure is shown in a schematic diagram (Figure 1B). Brifely, the mice were placed in an anesthesia induction chamber (Beijing Zhongshi Di Chuang Development Technology Co., Ltd., Beijing, China) pre-filled with 5% sevoflurane, and then removed when the reflexes disappeared, and fixed in prone position on a single-arm digital stereotaxic apparatus (Beijing Zhongshi Di Chuang Development Technology Co., Ltd., Beijing, China) To keep both eyes moist, vitamin A palmitate ophthalmic gel (Lot No.: 220101, Shenyang Xingqi Pharmaceutical CO., LTD., Shenyang, China) was applied to both eyes of the mice. Subsequently, the skin was prepared, disinfected, and the scalp was locally infiltrated with 0.5% lidocaine. The scalp was cut longitudinally and the periosteum was removed to expose the skull, followed by drilling a tiny hole 1.8 mm posterior to bregma at 1.2 mm on each side. The CA1 area of the hippocampus is reached by inserting glass electrodes along the small holes to a depth of 1.8 mm from the cortex. Subsequently 500 nl of rAAV5-GfaABC1D-hM3D(Gq)-mCherry (Titer: 3.05E + 12 VG/ml, BC-0476, Brain Case, China, Shenzhen) or rAAV5-GfaABC1D-mCherry (Titer: 3.25E + 12 VG/ml, BC-0908, Brain Case, China, Shenzhen) was injected at 50 nl/min. The needle was left in place for 10 min after injection, and the glass electrode was slowly withdrawn. Finally, the skull notch was closed with bone wax and the incision was sutured.

### Preparation of clozapine N-oxide

Clozapine N-oxide (CNO, S6887, Selleck Chemicals, USA) 50 mg was dissolved in 10 ml of dimethyl sulfoxide (DMSO, ST038, Beyotime, Shanghai, China) solution to prepare a CNO master mix at a concentration of 5 mg/ml. For intraperitoneal injection, CNO was diluted to 0.25 mg/ml working solution using saline. A dose of 2.5 mg/kg CNO was administered intraperitoneally 30 min before the behavioral test. The control solvent was saline containing the same dose of DMSO (subsequently referred to collectively as saline).

### Open field test

The open field tests were performed on mice in tibial fracture and control groups on postoperative day 7 to compare the locomotion of the mice (*n* = 10). The bottom surface of the open field box (40 cm × 40 cm × 40 cm) is equally divided by 16 quadrants, the middle 4 quadrants are the central area and the remaining 12 quadrants are the border area including the 4 corner quadrants that were are analyzed as the corner area. We cleaned the open field boxes with 75% alcohol before testing to avoid foreign smells that might influence mice behavior. After 10 s of adaptation, the behavior of mice was recorded for 5 min. Computerized tracking systems (Labmaze V3.0, Beijing Zhongshi Di Chuang Development Technology Co., Ltd., Beijing, China) were used to analyze the distance and speed of movements in mice and time they spent in specific regions.

### Elevated plus maze test

The elevated plus maze consisted of two relative open arms (25 cm long and 6 cm wide) and two relative closed arms (25 cm long, 6 cm wide and 20 cm high) and a central area (6 cm × 6 cm) connected. Mice were gently stroked in a quiet room for 5 min and then placed with their heads facing the open arm in the central area of the elevated maze. The number of times the mice entered the open arm and the residence time within 5 min were recorded using the animal behavior video analysis system (Labmaze V3.0, Beijing Zhongshi Di Chuang Development Technology Co., Ltd., Beijing, China), and the proportion of residence time in the open arm was calculated.

### Novel object recognition test

Mice were trained in novel object recognition on the 7th postoperative day (n = 10). Briefly, two identical, odorless, non-smooth, non-movable objects A and B were placed in symmetrical positions and the mice were allowed to explore them for 10 min for familiarization. Memory tests were performed on postoperative day 8. The familiar object B was replaced with a novel object C of a different shape, and then the mice were placed in a behavioral test chamber and their exploratory behavior was recorded for 5 min by a behavioral analysis system (Labmaze V3.0, Beijing Zhongshi Di Chuang Development Technology Co., Ltd., Beijing, China). Percentage of novel object exploration = novel object exploration time/(novel object exploration time + familiar object exploration time) × 100%; discrimination index = (novel object exploration time – familiar object exploration time)/(novel object exploration time + familiar object exploration time) × 100%.

### Fear conditioning test

Aging mice on day 10 post-anesthesia/surgery were subjected to 3 cycles of conditioned fear memory training with context-cue-electric shock pairing (n = 10). Briefly, mice were placed in a square test chamber with a white background for 180 s, then conditioned sound (70 dB, 3 kHz) was added for 30 s, followed by an electric shock (0.75 mA) for 2 s. The entire procedure was repeated three times. The freezing time for the first 180 s of placement in the conditioned fear box was recorded as a baseline. The chamber was wiped with 75% alcohol between each experiment to prevent residual odor from affecting the next experiment. The context association experiment and the cue association experiment were conducted separately on the second day. The context association test was performed as follows: the mice were placed in the same background test chamber for 180 s without adding sound stimuli, and the freezing time of the mice was recorded using the animal behavior analysis software (Labmaze V3.0, Beijing Zhongshi Di Chuang Development Technology Co., Ltd., Beijing, China). Two hours later, the cue association test was performed. The procedure was as follows: the test chamber was modified from a white background to a blue background, and the square activity space was modified to a triangular shape. Mice were placed in the modified new contextual chamber for 180 s. Mice were then subjected to a 180 s sound spike (70 dB, 3 kHz) and their freezing time during this period was recorded. The percentage of freezing time associated with context or with sound was compared between groups of mice to assess fear memory function.

### Golgi-cox staining

Three mice in each group were randomly selected for Golgi staining on day 10 after tibial fracture surgery (*n* = 3). Mice were executed under deep anesthesia with 8% sevoflurane, and brain tissues were fixed in 4% paraformaldehyde (G1101, Wuhan servicebio technology CO., Ltd., Wuhan, China) for 48 h. The brain tissues were cut into 2 mm thick pieces, gently rinsed several times with saline, and then placed in Golgi staining solution (G1069, Wuhan servicebio technology CO., Ltd., Wuhan, China) and changed to new staining solution every 3 days for 14 days in a cool and ventilated place. The brain tissue was then washed 3 times with double distilled water (ddH_2_O) and incubated overnight in 80% glacial acetic acid (10,000,218, Sinopharm Chemical Reagent Co., Ltd., Shanghai, China). The brain tissue was then rinsed with ddH_2_O and dehydrated in 30% sucrose. The brain tissue was cut into 100 μm sections using an oscillating microtome (CRYOSTAR NX50, Thermo, America) and then attached to gelatin slides. The air-dried tissue slides were treated with concentrated ammonia for 15 min, followed by ddH_2_O rinsing for 1 min and then treated with acidic firm film fixative for 15 min, ddH_2_O rinsing for 3 min and air-drying, and sealed with glycerol gelatin. Two microscopic fields were taken from each mouse in the hippocampus bilaterally and quantitative analysis was performed using Image-Pro Plus 6.0 software. The number and length of dendritic spines in the 30 ~ 90 μm length range on the second or third branch of the neuron were measured, and the density of dendritic spines per 10 μm was calculated according to the following formula: density = number of dendritic spines/dendritic length × 10. Ten concentric circles with 10 μm spacing centered were drawn on the cell body using the Sholl analysis plug-in, and the number of intersections between the dendrites and the concentric circles was calculated.

### Long-term potential recording

On day 11 after tibial fracture surgery, three mice per group were randomly selected for neurophysiological testing (*n* = 3). Briefly, 0.2% sodium pentobarbital 50 mg/kg was injected intraperitoneally and the mice were immobilized on a stereotaxic apparatus when the bracing reflex disappeared, then the head hair was removed after sterilization and the scalp was incised medially to expose the skull. Stimulating and receiving electrodes (Kedou Brain-Computer Technology, China, Suzhou) were implanted after drilling at two sites: (1) the Schaffer lateral branch site: anteroposterior (AP), −1.2 mm from Bregma, mediolateral (ML), −2.2 mm, dorsoventral (DV), 1.3 mm and (2) the granular cell layer site in the CA1 region: AP, −2.0 mm, ML, −1.5 mm, DV, 1.5 mm. Subsequent stimulation was divided into two phases to induce long-term potential. Phase I: Stimulation parameters were adjusted to a frequency of 1/60 Hz, a wave width of 100 s, and a current of 0.3 mA to induce a cluster peak potential. The stimulation electrode and recording electrode were then adjusted to obtain the optimal group spike (PS), and after 30 min of stabilization, the stimulation intensity was adjusted so that the PS was 1/3–1/2 of the maximum value and recorded for 30 min as a baseline; Phase II: the stimulation parameters were adjusted to 5 pulses of 400 Hz, repeated 3 times with 10 s interval, and then the PS was recorded for 120 min after high frequency stimulation (HFS). The slope of the averaged field excitatory postsynaptic potential (fEPSP) recorded for the last 20 min was used for analysis.

### Immunofluorescence staining of glial fibrillary acidic protein

Four mice in each group were randomly stained with immunofluorescence on day 11 after tibial fracture to observe the morphological and quantitative changes of astrocytes (*n* = 4). Briefly, paraffin sections were sequentially immersed in xylene and graded concentrations of alcohol to elute the paraffin, and subsequently placed in a modified sodium citrate reagent (P0083, Beyotime, Shanghai, China) and boiled for 20 min, then cooled to room temperature to repair the antigen. After washed thrice in PBS (C0221A, Beyotime, Shanghai, China), the sections were incubated with the quick block solution (P0222, Beyotime, Shanghai, China) for 1 h at room temperature. Sections were then washed again thrice in PBS before incubation with primary polyclonal rabbit antibody against GFAP (1:500, ab1872, Abcam, UK); primary polyclonal rabbit antibody against NEUN (1:200, ET1602-12, HuaBio, Hangzhou, China); primary polyclonal rabbit antibody against Iba-1 (1:200, AF7143, Beyotime, Shanghai, China) and mouse monoclonal antibody mCherry (1:150, K200015M, Solarbio, Beijing, China) overnight at 4°C. After washing thrice with PBS, the secondary antibodies cy3-conjugated goat anti-rabbit IgG (1:200, A5608, Beyotime, Shanghai, China) and FITC-conjugated goat anti-mouse IgG (1:200, A0568, Beyotime, Shanghai, China) was added to incubation for 1 h at room temperature. After washing thrice with PBS, DAPI (P0137, Beyotime, Shanghai, China) was added to stain the cell nuclei for 2 min to show their locations. Images were captured using a laser scanning microscope (Mingmei Photoelectric Technology Co., Ltd., Guangzhou, China). Two fields of visualization were taken from each hippocampal CA1 region on each side of each mouse for analysis, and the number of GFAP-labeled astrocytes under each visual field was counted. The fluorescence area of GFAP under each field of vision was measured using Image J (National Institutes of Health). Three astrocytes with relatively intact structure were selected in each field of view to calculate the fluorescence area.

### Statistical analysis

The software GraphPad Prism 9.0.1 (GraphPad Software, Inc.) was used to perform data analysis. The data were tested for normality using the Shapiro test, and normally distributed continuous variables were expressed as mean ± standard deviation. The difference between the C and TF groups were compared by unpaired *t*-tests and multiple comparisons using Bonferroni-corrected *p*-values. Correlation data for the three groups were analyzed using one-way ANOVA with a Tukey *post hoc* test for multiple comparisons. *p* < 0.05 was defined as a statistical difference.

## Results

### Decreased motor function and cognitive impairment occurred in aged mice after tibial fracture surgery

**Figure 2:**
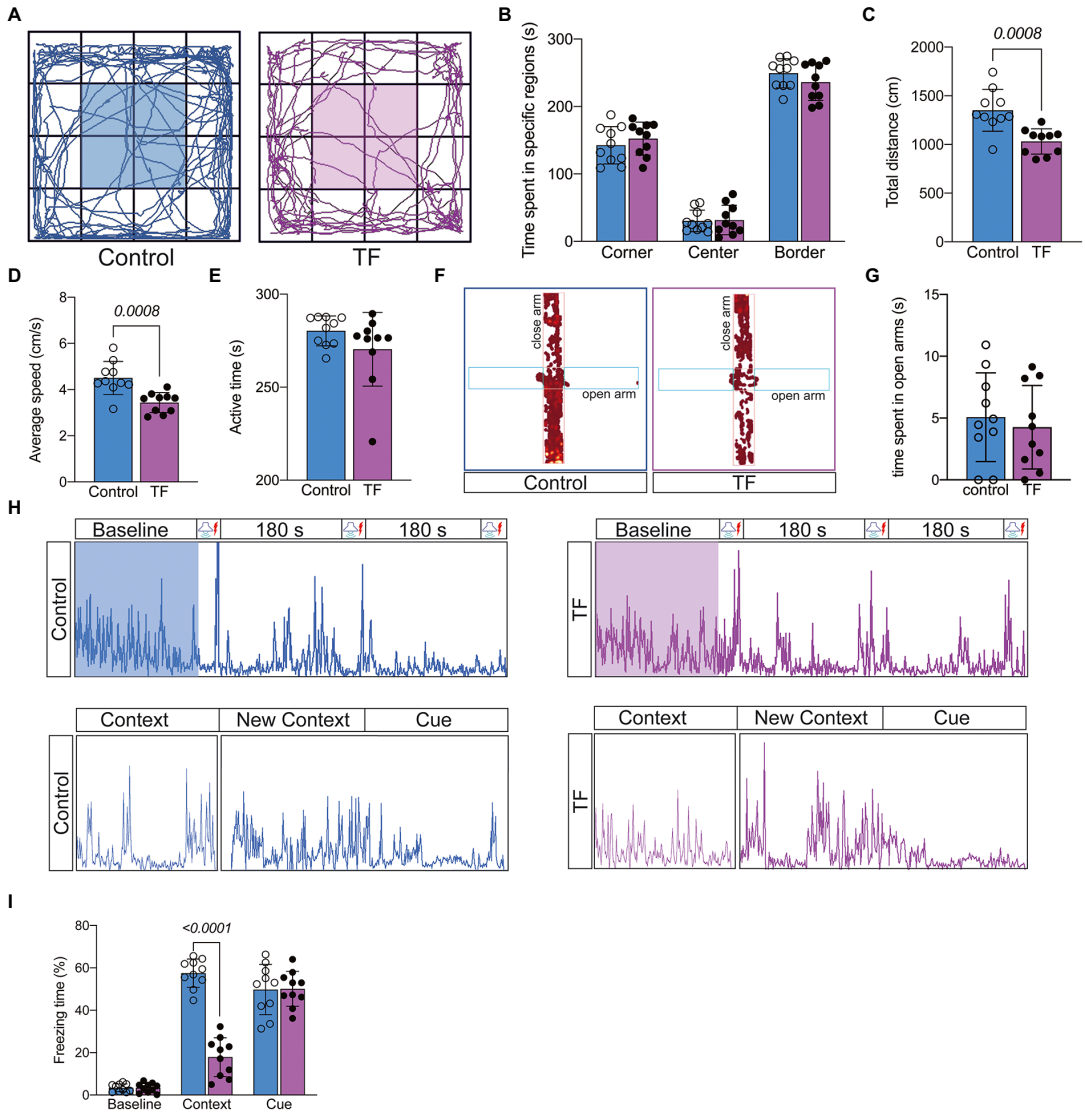
Behavioral testing of aged mice after tibial fracture surgery. **(A)** Mobility of aged mice was measured by the open-field test on the 7th day after fracture surgery. **(B–E)** Comparison of the results of the open-field test between the two groups of mice. **(F,G)** Anxiety in aged mice was measured using the elevated plus maze on day 7 after fracture surgery. **(H)** The image above shows the activity of mice trained in 3 cycles of Context (180 s)-Cue (30 s)-Shock (2 s) pairing on day 8 after fracture surgery. The image below shows the activity of the mice during the test phase on day 9 after fracture surgery: Context (180 s), Novel Context (180 s)-Cue (180 s). **(I)** Comparison of the percentage of freezing time in each phase for both groups of mice. Data were expressed as mean ± SD and analyzed using two-tailed unpaired *t*-test. The *p*-values are shown in the picture.

On day 7 after tibial fracture surgery, we tested the spontaneous mobility of the mice using the open field experiment (Figure 2A). To eliminate the effect of surgical factors, we performed X-ray examinations and did not detect any deformity healing (Supplementary Figure 1). The results of the open filed test showed no significant difference in activity time in the specific regions of the mice in the TF group compared to the C group (*P* > 0.05, Figure 2B), but the total distance traveled was reduced and locomotor speed decreased (**P* <* 0.05, Figures 2C,D), indicating that the mice had no significant emotional abnormalities but still had activity limitations 7 days after the anesthesia/surgery. To exclude the effect of postoperative anxiety, we further assessed the anxiety level of the mice with an elevated maze and found no significant difference between the two groups of mice (*P* > 0.05, Figures 2F,G; Supplementary Figure 2). Because of the difference in spontaneous activity between the two groups of mice (*p* > 0.05, Figure 1E), we excluded the usage of the Morris water maze and novel object cognition to assess the memory ability of the mice. We finally chosen the conditioned fear experiment, which is based on the Pavlovian conditioning principle and less dependent on activity ability, to assess memory function (Figure 2H). The results revealed no significant difference in freezing time between the two groups of mice at baseline and in the cue association test (*P* > 0.05, Figure 2I), while freezing time was significantly reduced in the context-related test (*P* > 0.05, Figure 2I). Taken together, these results indicate that tibial fracture surgery in aged mice leads to a hippocampus-dependent decline in cognitive function.

### Synaptic plasticity is altered in the hippocampal CA1 region of aged mice with POCD

**Figure 3:**
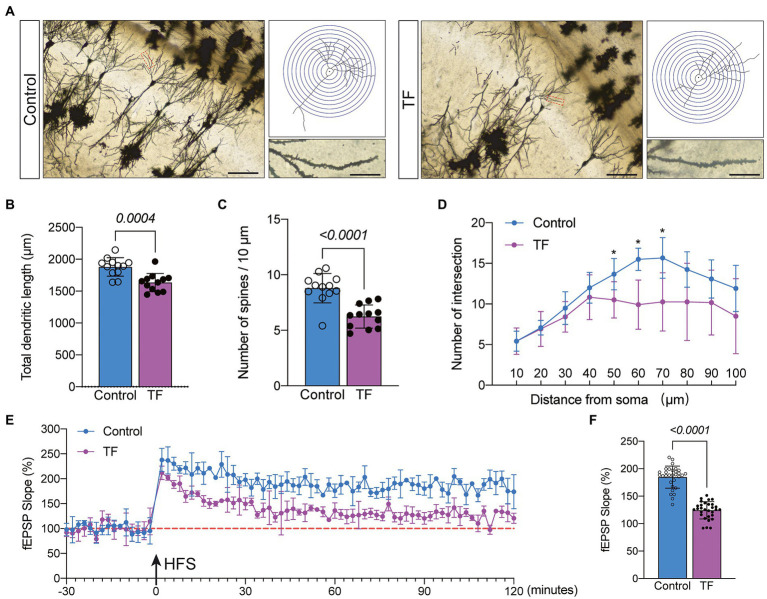
Synaptic plasticity in hippocampal CA1 region at day 10 after fracture surgery. **(A)** Golgi staining of the CA1 region of the mouse hippocampus (×200, *n* = 3), Sholl analysis and magnification of synapses (×1,000), scale bar = 100 or 20 μm. **(B–D)** Quantitative analysis of total synaptic length, synaptic spine density and synaptic number. The Number of intersection was analyzed using unpaired *t*-test with Bonferroni corrected *p*-values. **(E)** Recording and analysis of local field potentials in the CA1 region of the hippocampus in response to stimulation of Schafer’s lateral branch fibers (*n* = 3). **(F)** Average fEPSP Slop in the last 20 min. *p < 0.05, compared with the C group. Data are presented as mean ± SD. Data were expressed as mean ± SD and analyzed using two-tailed unpaired *t*-test. The *p*-values are shown in the picture.

To further determine the memory function in mice, we performed Golgi staining of the hippocampal CA1 brain region responsible for short-term memory to observe the changes in synaptic structure (Figure 3A). The dendritic length, dendritic and dendritic spine density of neurons in the hippocampal CA1 region were quantified by the Sholl analysis method in Image J. The results showed that the total dendritic length (**P* <* 0.05, Figure 3B), dendritic spine density (**P* <* 0.05, Figure 3C), and the intersection of dendrites with concentric circles (**P* <* 0.05, Figure 3D) were reduced in the hippocampal CA1 neurons of the TF group compared with the C group. This indicates that the precise and complex structure of the dendrites of neurons in the CA1 region of the hippocampus of mice in the TF group has changed. We further assessed the synaptic plasticity of neurons by examining the slope of mean field excitatory postsynaptic potentials (fEPSP) in the CA1 region of the mouse hippocampus (Figure 3E). The results showed that the decreased LTP in the CA1 brain region of aged mice undergoing tibial fracture surgery indicated that synaptic plasticity was functionally impaired in the TF group of mice (**P* <* 0.05, Figure 3F).

### Increased abnormal activation of astrocytes in the CA1 region of the hippocampus in POCD-aged mice

**Figure 4:**
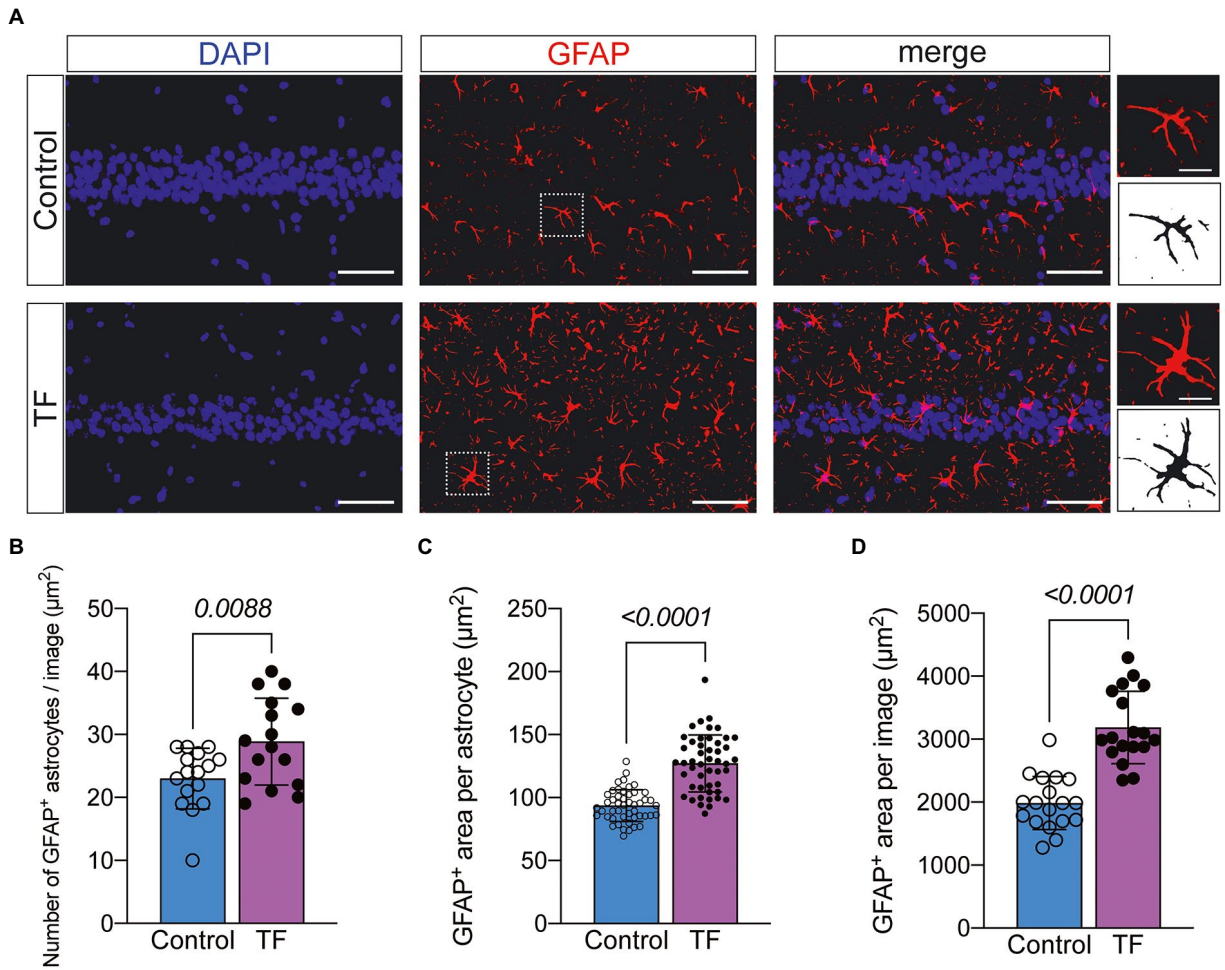
Immunofluorescence staining of astrocytes on day 10 after fracture surgery. **(A)** GFAP-labeled astrocytes in the hippocampal CA1 region (*n* = 4). Scale bar = 50 μm or 10 μm. **(B–D)** Quantitative analysis of GFAP^+^ astrocytes. Data were expressed as mean ± SD and analyzed using two-tailed unpaired *t*-test. The *p*-values are shown in the picture.

To further investigate the possible causes of impaired synaptic plasticity in neurons in the CA1 region of the hippocampus of aged mice with POCD, we observed morphological changes in astrocytes that are actively involved in maintaining synaptic function. We labeled astrocytes with glial fibrillary acidic protein (GFAP) and found increased abnormal activation of astrocytes in the hippocampal CA1 region of aged mice undergoing anesthesia and surgery (Figure 4A). The results of immunofluorescence revealed an increased number of GFAP+ astrocytes in the hippocampal CA1 region of mice in the TF group compared to the C group (**P* <* 0.05, Figure 4B), an increased area of GFAP+ fluorescence staining per field of view (**P* <* 0.05, Figure 4C), and an increased area of fluorescence of individual glial cells (**P* <* 0.05, Figure 4D).

### Gq-pathway activation of astrocytes in hippocampal CA1 region can improve cognitive dysfunction caused by anesthesia/surgery

**Figure 5:**
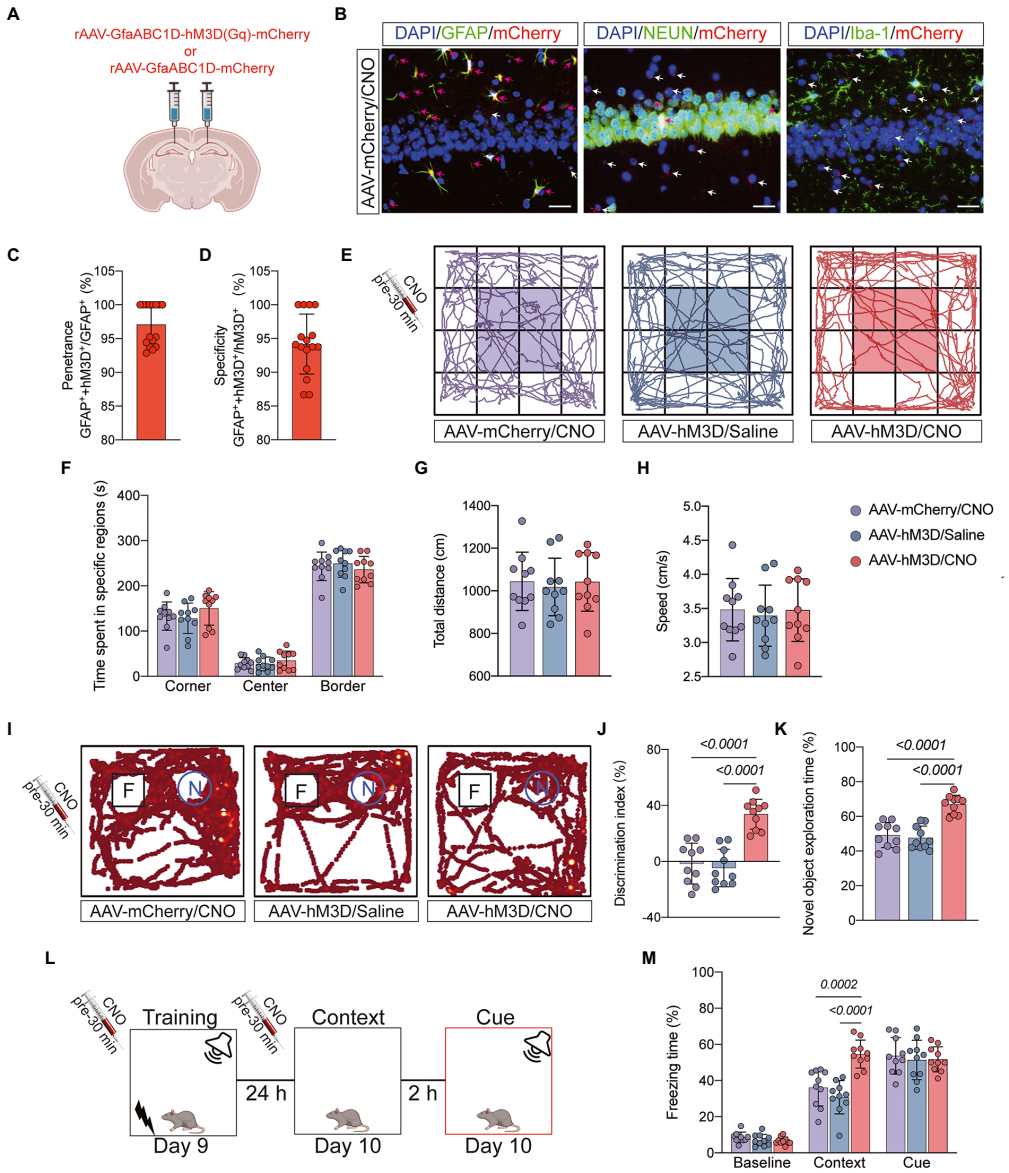
Behavioral effects of Gq pathway activation in astrocytes of hippocampal CA1 region. **(A)** Bilateral hippocampal CA1 regions were injected with rAAV-GfaABC1D-hM3D(Gq)-mCherry (*n* = 20) or rAAV-GfaABC1D-mCherry (*n* = 10) 21 days before surgery. **(B)** hM3D(Gq) was specifically expressed in astrocytes. GFAP was used to label astrocytes, NEUN to label neurons, Iba-1 to label microglia, mCherry to label hM3D(Gq), and DAPI to label nuclei, respectively. Red arrows indicate double-staining positivity, while white arrows indicate mCherry single-staining positivity. Scale bar = 25 μm. **(C)** The ratio of GFAP and mCherry double-positive cells to GFAP-positive cells is the epistasis rate. **(D)** The ratio of GFAP and mCherry double-positive cells to mCherry-positive cells is the specificity rate. **(E)** Locomotor activity was measured on day 7 after tibial fracture by open-field assay after 30 min of intraperitoneal injection of CNO or saline. **(F–H)** Quantification of total distance travelled, speed of movement, and time spent moving in specific areas in 3 groups of mice. **(I)** Novel object recognition tests were performed on the 7th and 8th day after tibial fracture. **(J,K)** Quantitative analysis of discrimination index and percentage of new object recognition in the 3 groups of mice. **(L,M)** Three cycles of training were performed on postoperative day 9, and the percentage of freezing time was assessed on day 10 in the Context test and the Cue test. Data were expressed as mean ± SD and analyzed by *ANOVA* test with a Tukey *post hoc* test. The *p*-values are shown in the picture.

Astrocytes can exchange information with neurons, respond to synaptic activity, and regulate synaptic transmission. Abnormal activation of astrocytes is often accompanied by functional abnormalities. To verify that abnormal activation and decreased function of astrocytes in the CA1 region are important factors contributing to POCD, we injected the chemical genetic reagent rAAV-GfaABC1D-hM3D(Gq)-mCherry (AAV-hM3D/CNO) into the bilateral hippocampal CA1 region of aged mice 3 weeks before TF surgery to specifically activate hippocampal astrocytes in the CA1 region (Figures 5A,B). At 7 days after TF surgery, the locomotor activity of the mice was detected by open field test, and there was no difference between the three groups (*P* > 0.05, Figures 5C–F). Subsequently, we examined the memory function of the mice with novel object recognition and fear conditioning test. The results showed that mice in the AAV-hM3D/CNO group spent more time exploring the new object and showed more freezing time when recalling context-related fear memories compared to the AAV-mCherry/CNO and AAV-hM3D/Saline groups (**P* <* 0.05, Figures 5G–K). In contrast, the total distance of movement and average movement speed did not differ among the 3 groups of mice (*P* > 0.05, Supplementary Figure 3). These results suggest that Gq pathway activation in astrocytes of hippocampal CA1 area can improve cognitive dysfunction induced by anesthesia/surgery.

### Gq pathway activation of CA1 area astrocytes ameliorates anesthesia/surgery induced cognitive dysfunction associated with enhanced synaptic plasticity

**Figure 6:**
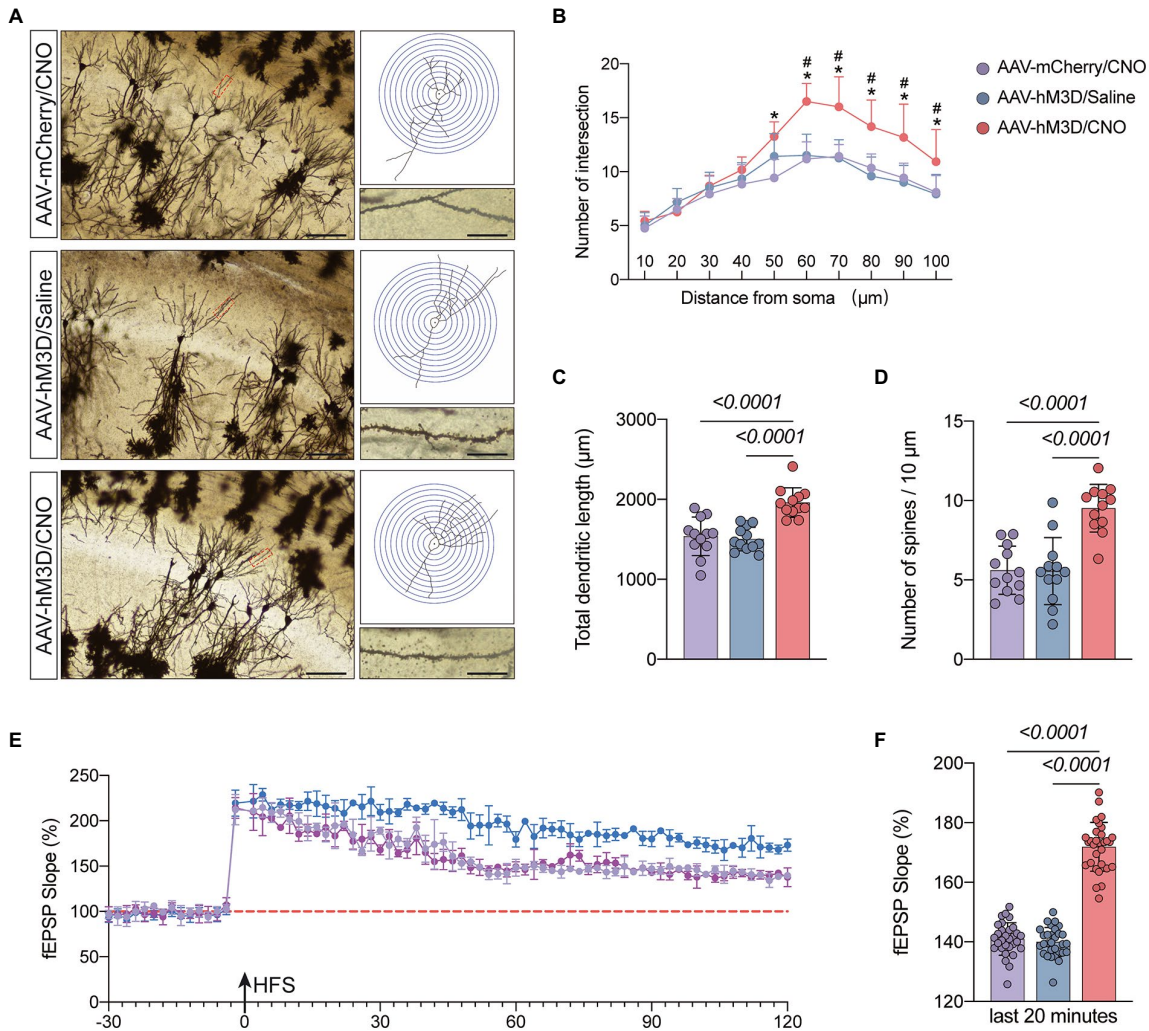
Effects of activation of Gq pathways in astrocytes on the structure and function of synaptic plasticity at postoperative day 11. **(A)** Golgi staining of the CA1 region of the mouse hippocampus (×200, *n* = 3), Sholl analysis and magnification of synapses (×1,000), scale bar = 100 or 20 μm. **(B–D)** Quantitative analysis of total synaptic length, synaptic spine density and synaptic number. **(E)** Recording and analysis of local field potentials in the CA1 region of the hippocampus in response to stimulation of Schafer’s lateral branch fibers (*n* = 3). **(F)** Average fEPSP Slop in the last 20 min. *p < 0.05, compared with the AVV-mCherry/CNO group. #*p* < 0.05, compared with the AVV-hM3D/Saline group. Data were expressed as mean ± SD and analyzed by *ANOVA* test with a Tukey *post hoc* test. The *p*-values are shown in the picture.

We hypothesized that activation of the Gq pathway in astrocytes might be associated with improved synaptic plasticity. To confirm this, we observed synaptic structures by Golgi staining (Figure 6A). The results demonstrated that mice in the AAV-hM3D/CNO group had increased total dendritic length, dendritic density and dendritic spine density compared to the AAV-mCherry/CNO and AAV-hM3D/Saline groups (**P*<*0.05, Figures 6B–D). We further assessed synaptic function by monitoring fEPSP and found that mice in the AAV-mCherry/CNO group had significantly increased fEPSP compared to the AAV-mCherry/CNO and AAV-hM3D/Saline groups (**P*<*0.05, Figures 6E,F). The above results indicate that Gq pathway activation in astrocytes can improve synaptic plasticity of neurons structurally and functionally.

## Discussion

**Figure 7:**
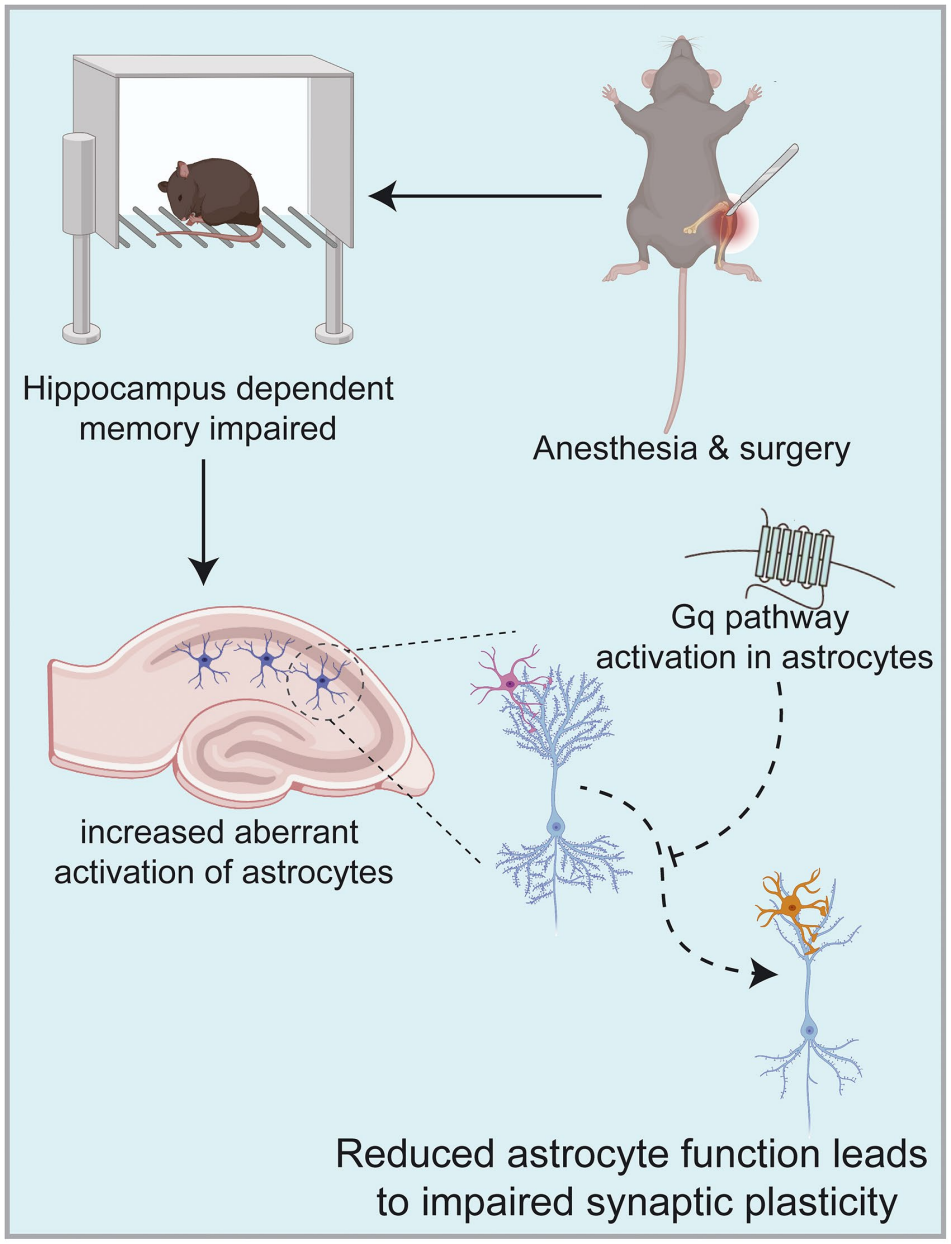
Anesthesia/surgery in aged mice can result in hippocampus-dependent cognitive dysfunction accompanied by altered synaptic plasticity in hippocampal CA1 regions. This mechanism may be related to increased aberrant activation of astrocytes in the hippocampus, among which astrocytes are known to be active participants in synaptic processing. In contrast, activation of Gq pathways in hippocampal astrocytes improves hippocampal-dependent cognitive dysfunction. Images were created on the BioRender web platform.

Our study aimed to determine that astrocytes are important players in learning memory and that the occurrence of POCD in elderly patients may be related to their abnormal function. Our results suggest that activation of the astrocyte Gq pathway improves learning memory in POCD-aged mice. Astrocytes are known to be active participants in synaptic processing, and their functional state influences synaptic structure and function. Our findings suggest that the occurrence of POCD in aged mice is accompanied by abnormal astrocyte activation and altered synaptic plasticity. In contrast, activation of the Gq pathway in astrocytes using a chemical genetic approach improves learning memory capacity. This was evidenced by increases in dendritic length, number, branching and synaptic spine density; increases in LTP strength; enhanced fear memory in fear conditioning test and increased time to explore new objects in novel object recognition test. Overall, astrocytes can be used as a target for the prevention and treatment of POCD in elderly patients (Figure 7).

With the aging of the population and the development of medical technology, more and more elderly fracture patients are given the opportunity to undergo surgery (Tzimas et al., 2018). POCD, a common complication after surgery for geriatric fractures, not only prolongs hospitalization and increases treatment costs, but also increases the occurrence of other related complications and even increases the postoperative mortality of patients (Zhu et al., 2021). Therefore, it is important to prevent and reduce the occurrence of POCD. In this study, we used 18-month-old mice to perform tibial osteotomy with intramedullary nail fixation under sevoflurane anesthesia to simulate the anesthetic surgical procedure in clinically aged patients. Postoperatively, we performed x-rays to ensure that the tibial fracture was anatomically repositioned (Supplementary Figure 1). On postoperative day 7, we examined the ability of the mice to move autonomously using the open field test, which showed that although the mice had achieved anatomical repositioning, they still had limited motor function. This may lead to unreliable results in either the Morris water maze or the novel object recognition test. This is inconsistent with the results of Qiu et al.’s (2020) study, which may be caused by our choice of older mice with poorer recovery, but the use of older mice for modeling is more clinically relevant. How to scientifically and correctly detect the memory function of mice after surgery troubled us, so we re-analyzed the results of open field test and found that although the motor speed and motor distance were reduced in older mice after TF surgery, their active time or immobility time was consistent (Figure 1E). Therefore, we chose the fear conditioning test, which is based on the Pavlovian effect and less dependent on motor ability, to test memory function. The results showed that the freezing time baseline in the training phase of the mice in the TF and C groups was consistent. This indicates that it is scientifically feasible to compare the memory function of two groups of mice by fear conditioning test. The results of the recall phase demonstrated that the freezing time associated with the context decreased significantly in the older mice after TF surgery, while there was no difference in the freezing time associated with the cue. Hippocampal-amygdala circuits are the core of contextual fear associated memory (Liu et al., 2012; Lacagnina et al., 2019). Cue-related fear memory is established mainly by auditory cortex and auditory thalamus-amygdala neural circuits (Choi, D. I., et al., 2021). Based on these theories and the results we obtained, it is implied that hippocampus-dependent memory dysfunction occurs in aged mice that have experienced anesthesia and surgery. These results are consistent with the experimental results of Lin et al. (2021). It fully illustrates that sevoflurane anesthesia combined with TF surgery successfully built POCD model.

The hippocampus is central to the cognitive functions, and there is growing evidence that POCD is associated with impaired synaptic transmission, reduced neurotransmitters, neuronal apoptosis and altered synaptic plasticity in the hippocampus (Erickson et al., 2011; Chen et al., 2019; Zheng et al., 2019). Reduced dendritic spine and synaptic is characteristic of many neurological disorders, including autism spectrum disorders, schizophrenia, and Alzheimer’s disease (Han et al., 2012; Reddy et al., 2018; Stampanoni Bassi et al., 2019). Recent studies have shown that POCD is accompanied by impaired synaptic plasticity in the CA1 region of the hippocampus (Chen et al., 2020). Our Golgi staining results showed reduced synaptic density and reduced number of dendritic spines in the hippocampal CA1 region of POCD mice. The neurophysiological results showed reduced LTP amplitude and frequency in the hippocampal CA1 region of POCD mice. These results support the above theory in terms of synaptic structure and synaptic function. Astrocytes are morphologically complex cells that comprise approximately half of the brain cells and are involved in a variety of structural, metabolic, and homeostatic functions (Chen et al., 2020; Corkrum et al., 2020; Wang, J., et al., 2021). Glial cells are important regulators of synaptic function and plasticity. Astrocytes are a major class of glial cells that respond to neuronal activity by intracellular Ca^2+^ transients and then release gliotransmitters that in turn alter synaptic connections. This reciprocal neuron astrocyte interaction has led to the concept of the ‘tripartite synapse’, in which astrocytes are considered active participants in synaptic processing (Lee et al., 2021; Wang, Y., et al., 2021). The fine structure of well-developed astrocytes is highly plastic and activity-dependent (Habib et al., 2020; Choi, M., et al., 2021). They exhibit heterogeneity and morphological diversity in different brain regions, which may be related to astrocyte-neuron spatiotemporal interactions, which also supports the ‘tripartite synapse’ hypothesis (de Tredern et al., 2021; Sadick et al., 2022). Reactive astrocytes are marked by morphological alterations as well as defects in fine structure, which may drive disease progression and are a common pathological feature of many neurological disorders (Bondi et al., 2021). In the present study we used glial fibrillary acidic protein (GFAP) to label astrocytes and found an increase in GFAP-positive glial cells in the hippocampal CA1 region of POCD-aged mice, with expanded cell body and thickened branches. Study of Wang et al. (2022) had similar results that astrocyte morphology was altered and accompanied by impaired calcium signaling in POCD mice. We hypothesize that the occurrence of POCD is associated with altered morphological and biophysical properties of astrocytes resulting in reduced execution of synaptic modifications. Adamsky et al. (2018) expressed the Gq-coupled receptor hM3Dq in CA1 astrocytes in normal mice by chemical genetic techniques, allowing their activation by CNO. Gq pathway activation in astrocytes was found to lead to enhanced memory recall. Based on this study, we wanted to investigate whether activating astrocytes in POCD mice could improve cognition. We used chemical genetic tools in combination with microinjection of specific brain regions and controlled activation of the Gq-pathway by the GfaABC1D promoter to specifically manipulate astrocytes around hippocampal CA1. The mCherry as a red fluorescent reporter protein, is used for proper visualization of hM3D(Gq). We designed two control groups, the AAV-mCherry/CNO group and the AAV-hM3D/Saline group, in order to exclude the effects of CNO and viral particles. We injected AAV-mCherry or AAV-hM3D in the bilateral hippocampal CA1 region of the mice 21 days before surgery, and on the seventh postoperative day we assessed the motor ability of the mice and found no difference in the motor ability of the three groups. Therefore, we added a novel object recognition test to the memory assessment to make the results more convincing. CNO or saline was injected intraperitoneally 30 min before the training phase and testing phase of novel object recognition test and fear conditioning test. The results showed that the memory function of mice in the AAV-hM3D/CNO group was significantly improved compared with the AAV-mCherry/CNO group and the AAV-hM3D/Saline group. This might be related to the enhanced synaptic plasticity by the activation of Gq pathways in astrocytes. We then used Golgi staining technique and neurophysiological technique to judge synaptic plasticity structurally and functionally, respectively. The results showed a significant increase in dendritic density and number of dendritic spines in the AAV-hM3D/CNO group of mice, and a significant increase in LTP amplitude. This data suggests that the activation of astrocyte Gq pathways improved the function of astrocytes in modifying neuronal synapses, which is consistent with the findings of Van Den Herrewegen et al. (2021) and Kol et al. (2020). The aberrant activation of astrocytes may be the driving force for POCD production. The above results provide a basis for targeting astrocyte cells for clinical prevention and treatment of POCD.

However, there are still some limitations of our study. We only studied memory function between 7 and 11 days after anesthesia/surgery in aged mice and did not verify the effect on their long-term memory. We gave CNO during both the postoperative learning phase and the testing phase, so the specific effects of astrocyte Gq pathway activation on memory formation, memory consolidation or memory extraction remain to be further investigated. In this study, although we detected the expression of hM3D(Gq), no experiments related to the observation of changes in astrocyte calcium activity using two-photon microscopy or miniscop were performed. Direct evidence for Gq pathway activation is lacking. The activation of the Gq pathway in astrocytes involves a wide range of molecular signaling pathways, and the molecular mechanisms that play a major role need to be further investigated.

## Conclusion

Overall, our results suggest that tibial fracture surgery in aged mice can lead to recent hippocampal-dependent cognitive dysfunction. Impaired synaptic plasticity due to abnormal activation of astrocytes is an important mechanism for the occurrence of POCD. In contrast, activation of the Gq pathway of astrocytes in the CA1 region of the hippocampus ameliorates POCD in aged mice caused by aging anesthesia/surgery.

## Data availability statement

The original contributions presented in the study are included in the article/Supplementary materials, further inquiries can be directed to the corresponding author.

## Ethics statement

The animal study was reviewed and approved by Ethics Committee of the Third Hospital of Hebei Medical University.

## Author contributions

QW and XW conceived the idea of the study. ZH and FX analyzed the data. JZ and YZ interpreted the results. JY and QZ prepared the model and injected the virus into CA1 brain region. XW wrote the paper. QZ and CY conducted behavioral related test. All authors contributed to the article and approved the submitted version.

## Funding

This work was supported by grants from the *Key project of Precision Medicine Joint Fund of Hebei Natural Science Foundation* (H2021206021), and the *Hebei Provincial government funded the provincial Medical Talents Project*.

## Conflict of interest

The authors declare that the research was conducted in the absence of any commercial or financial relationships that could be construed as a potential conflict of interest.

## Publisher’s note

All claims expressed in this article are solely those of the authors and do not necessarily represent those of their affiliated organizations, or those of the publisher, the editors and the reviewers. Any product that may be evaluated in this article, or claim that may be made by its manufacturer, is not guaranteed or endorsed by the publisher.
