# Supplementary figures and images for: Activation of astrocyte Gq pathway in hippocampal CA1 region attenuates anesthesia/surgery induced cognitive dysfunction in aged mice
Source: Front Aging Neurosci. 2022 Nov 11;14:1040569. doi: 10.3389/fnagi.2022.1040569 (PMC9692004; doi:10.3389/fnagi.2022.1040569)

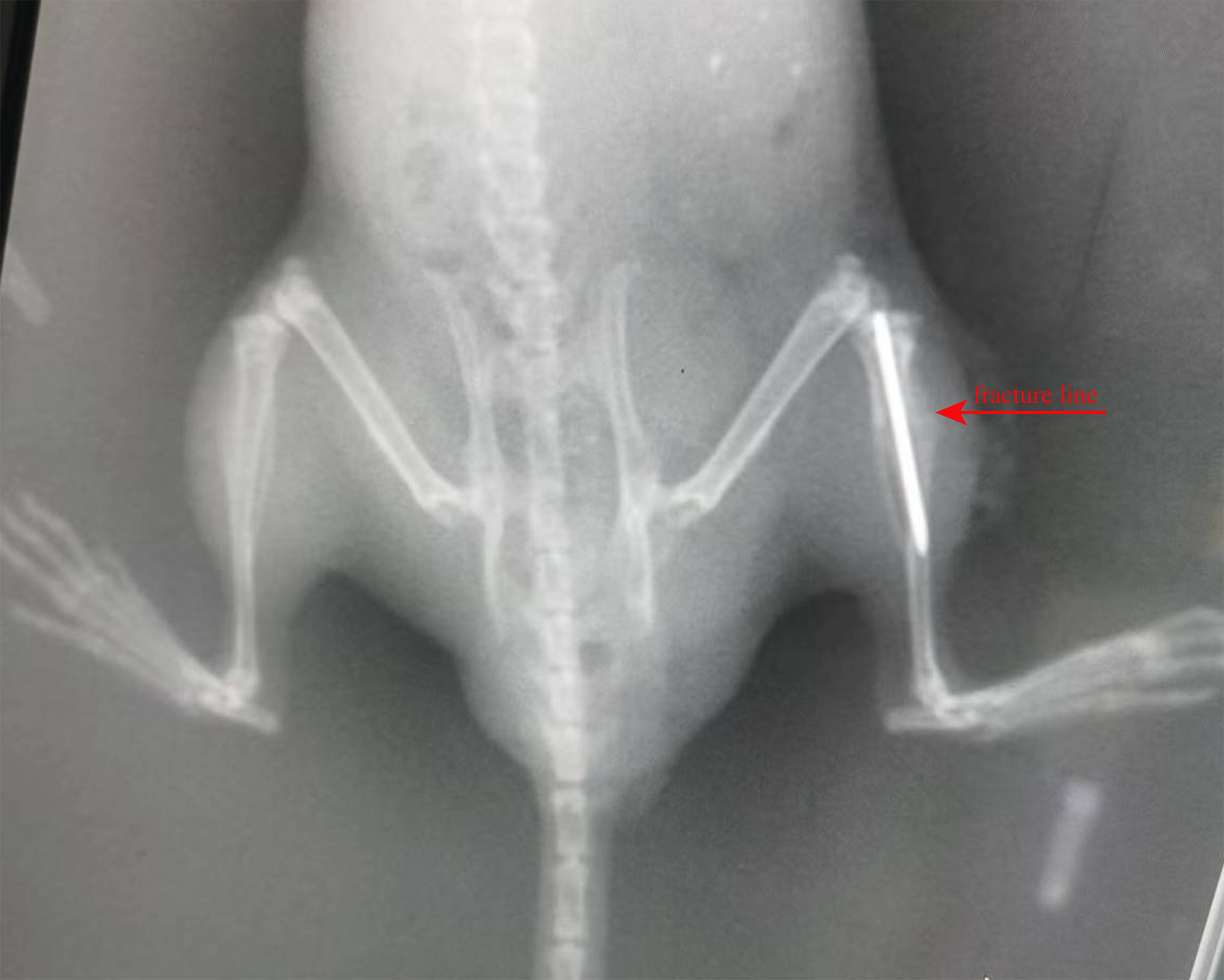

Supplement: Supplementary file 1 [file Image_1.TIF]

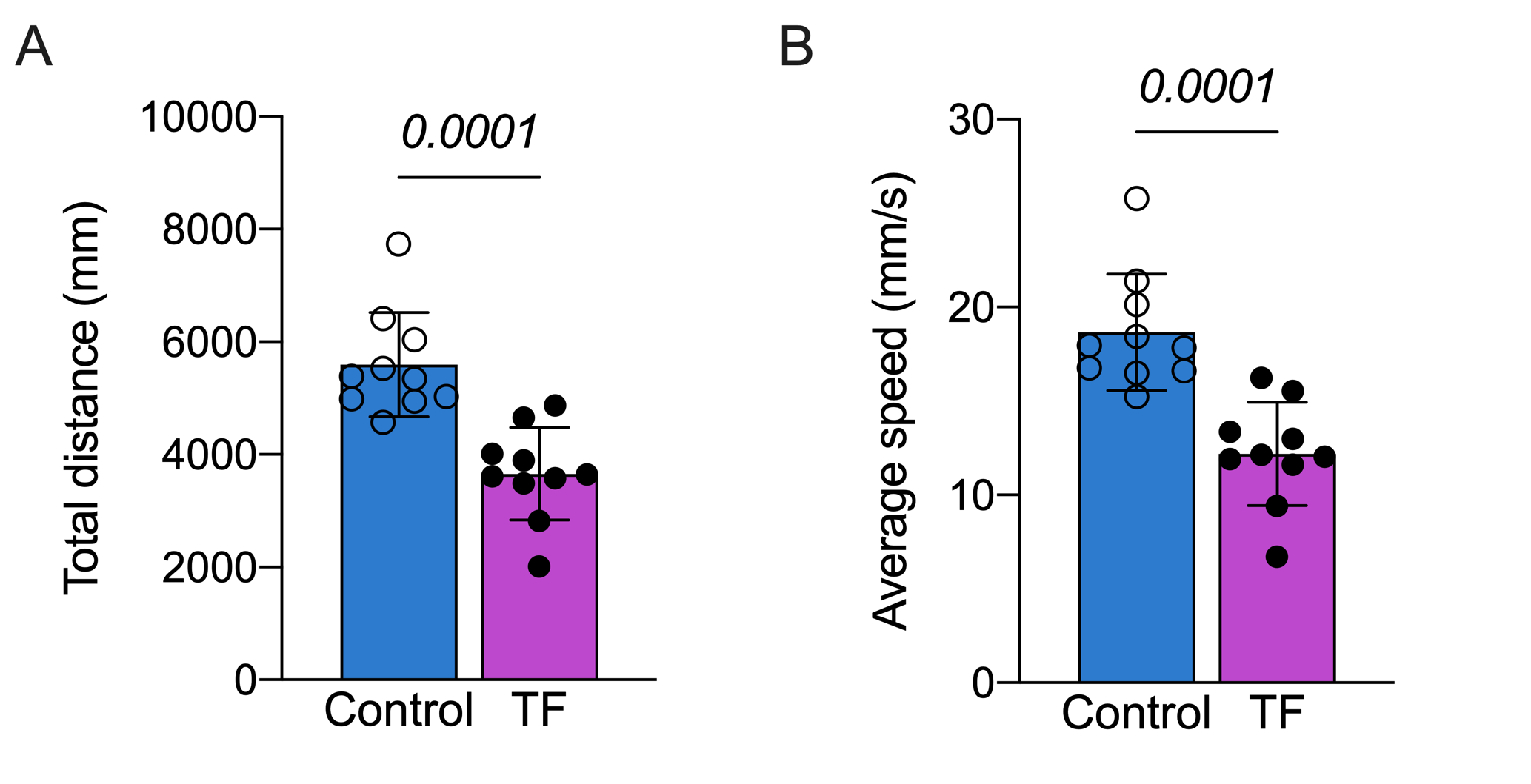

Supplement: Supplementary file 2 [file Image_2.TIFF]

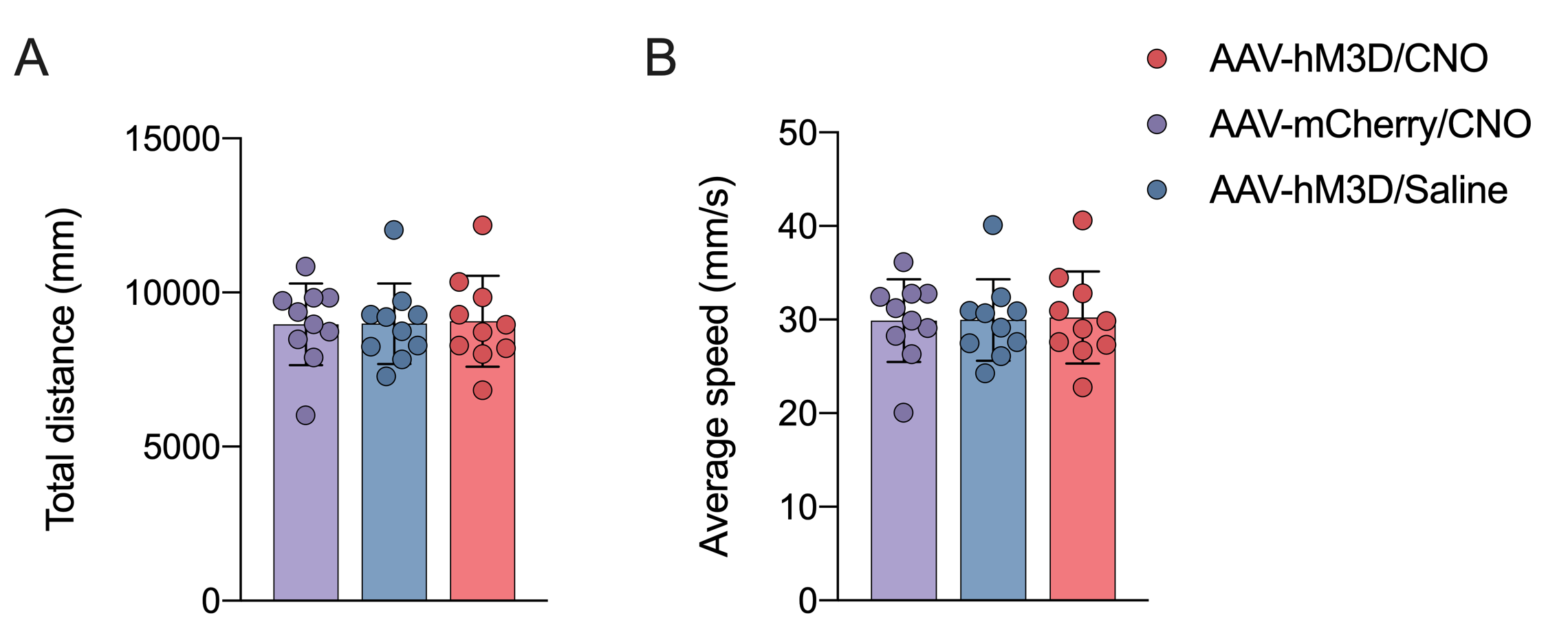

Supplement: Supplementary file 3 [file Image_3.TIFF]
